# Spatially-Explicit Simulation Modeling of Ecological Response to Climate Change: Methodological Considerations in Predicting Shifting Population Dynamics of Infectious Disease Vectors

**DOI:** 10.3390/ijgi2030645

**Published:** 2013-07-22

**Authors:** Radhika Dhingra, Violeta Jimenez, Howard H. Chang, Manoj Gambhir, Joshua S. Fu, Yang Liu, Justin V. Remais

**Affiliations:** 1 Department of Environmental Health, Rollins School of Public Health, Emory University, 1518 Clifton Rd. NE, Atlanta, GA 30322, USA; 2 Department of Biostatistics and Bioinformatics, Rollins School of Public Health, Emory University, 1518 Clifton Rd. NE, Atlanta, GA 30322, USA; 3 MRC Centre for Outbreak Analysis and Modeling, Department of Infectious Disease Epidemiology, Imperial College London, London, SW7 2AZ, UK; 4 Department of Civil and Environmental Engineering, University of Tennessee, Knoxville, 62 Perkins Hall, Knoxville, TN 37996, USA; 5 Program in Population Biology, Ecology and Evolution, Graduate Division of Biological and Biomedical Sciences, Emory University, 1510 Clifton Rd., Atlanta, GA 30322, USA

**Keywords:** vector-borne disease, spatially-explicit, dynamic, population model, *Ixodes scapularis*, climate change, temperature, population response, deer ticks

## Abstract

Poikilothermic disease vectors can respond to altered climates through spatial changes in both population size and phenology. Quantitative descriptors to characterize, analyze and visualize these dynamic responses are lacking, particularly across large spatial domains. In order to demonstrate the value of a spatially explicit, dynamic modeling approach, we assessed spatial changes in the population dynamics of *Ixodes scapularis*, the Lyme disease vector, using a temperature-forced population model simulated across a grid of 4 × 4 km cells covering the eastern United States, using both modeled (Weather Research and Forecasting (WRF) 3.2.1) baseline/current (2001–2004) and projected (Representative Concentration Pathway (RCP) 4.5 and RCP 8.5; 2057–2059) climate data. Ten dynamic population features (DPFs) were derived from simulated populations and analyzed spatially to characterize the regional population response to current and future climate across the domain. Each DPF under the current climate was assessed for its ability to discriminate observed Lyme disease risk and known vector presence/absence, using data from the US Centers for Disease Control and Prevention. Peak vector population and month of peak vector population were the DPFs that performed best as predictors of current Lyme disease risk. When examined under baseline and projected climate scenarios, the spatial and temporal distributions of DPFs shift and the seasonal cycle of key questing life stages is compressed under some scenarios. Our results demonstrate the utility of spatial characterization, analysis and visualization of dynamic population responses—including altered phenology—of disease vectors to altered climate.

## 1. Introduction

Understanding the ecological response to anthropogenic environmental changes, including changes in climate, land use, land cover and other factors, requires quantitative tools to characterize, analyze and visualize dynamic changes in the populations of key organisms of interest. Developing such tools is made difficult by the fact that population responses to environmental change are spatially and temporally complex, particularly for organisms with multiple environmental life stages, such as those that participate in the transmission of vector-borne diseases (VBD). Disease vector populations may exhibit variations in seasonal timing and duration, and their generally non-linear response to environmental signals makes prediction of the risk posed by VBD under altered environmental conditions challenging [1–3]. When exposed to changing climatic conditions, vector distribution and the risks of VBD may shift substantially across time and space [1,4,5]. Yet, a great deal of uncertainty remains for many VBD systems [4,6–8], and little is known regarding the dynamic nature of the population response to climate change, particularly vector phenology (timing of life stages), seasonality and the duration of key population events.

While some ecological analyses have characterized the dynamic population response of various plant and arthropod species to external forcings in a spatially explicit fashion (e.g., [9–12]), much analogous work on VBD has neglected the spatial domain [13,14]. Still, other work forgoes system dynamics, instead investigating the spatial patterns of static population measures, such as presence/absence or mean abundance (see, for instance, [15] for Lyme disease and [16] for hantavirus). Such analyses make use of statistical relationships between climate and habitat suitability to estimate, for instance, the potential changes in the distribution of habitat suitability for, or nymphal density of, *Ixodes scapularis*, the vector of Lyme disease [17,18]. This approach offers little insight into the nature of the population’s response over time, such as shifts in peak population timing or variability in population density during key exposure periods (e.g., high season for recreational activities). Given the substantial and continuing disagreement regarding how climate may change the distribution of VBD (e.g., [4,7]), analyses capable of assessing the relationship between exogenous forcings and population dynamics in space and time may provide such insights.

What is more, geovisualization of the dynamic VBD response to environmental change could provide key information (e.g., maps summarizing complex spatio-temporal phenomena) for developing policies to respond to shifting risk. Thus, geospatial tools for characterizing, analyzing and predicting the response of VBD to future changes are desirable, and these should emphasize dynamic phenomena known to be important for understanding risk, such as vector phenology and seasonality. Phenology—the timing of life stages—is known to be sensitive to climatic change and is an important determinant of the spatial distribution of arthropods [19,20]. Current models investigating arthropod distribution under future climates generally ignore phenology, instead, establishing a relationship between a vector’s current abundance and key habitat characteristics and, then, applying that model to projected future conditions [21,22]. An examination of an organism’s phenological response can reveal important, but subtle, impacts of changing climate. For instance, the date of flowering and fruiting have been shown to be important determinants of aspen distribution [23], and the date of first oviposition has been shown to be important for gypsy moth distribution [24]. Characterization of life stage-specific dynamic responses can highlight such subtle determinants of the distribution of vectors under the future climate.

The seasonality of events may also shift under future conditions, with important consequences for VBD risk. For instance, vector populations may peak at certain times of the year, with peak incidence of disease occurring at other times (e.g., see [25] for Lyme disease). Some models of VBD response to climate change attempt to roughly characterize changes in seasonality (e.g., [26]); some integrate seasonal elements, such as temperature, humidity and daytime hours, through degree-day models (e.g., [27–30]), and still, others do not explicitly account for seasonality (e.g., [31]). A more detailed spatial representation of seasonal shifts would make it possible to characterize the potentially profound effect that environmental change may have on the length and timing of VBD transmission seasons.

Here, we develop a spatially-explicit modeling approach for investigating the dynamic population responses of a disease vector of interest, with the goal of enhancing our understanding of future VBD risk. We introduce the concept of dynamic population features (DPFs), which provide information on population cycling, seasonal timing and phenological events across vector life stages. Importantly, we describe how analysis of such features—such as number and timing of population peaks (Table 1)—may be used to predict disease risk.

To demonstrate the utility of this modeling approach, we examine the responses of the black-legged deer tick (*Ixodes scapularis*), the vector for Lyme disease, to changes in temperature across the eastern United States. *I. scapularis* is an excellent model organism with which to examine the influence of climate change on phenological and seasonal characteristics: it is known to be highly sensitive to environmental conditions, including temperature [6,15,25,32]. Furthermore, the three *I. scapularis* life stages (larva, nymph and adult) require different temperature conditions to support host finding or progress to the next life stage [28].

We explore the Lyme disease system as a case study, simulating *I. scapularis* population dynamics over the eastern United States using modeled climate data, and spatially characterize, analyze and visualize key DPFs for each tick life stage. We examine DPFs from simulated dynamics under current climate conditions and compare these to observed data to ascertain which features best predict current levels of disease risk. We then project DPFs under two future climate scenarios and provide key geovisualizations of projected vector dynamics over the spatial range. We show, by characterizing and visualizing DPFs, how we can determine which population features best predict disease risk under current conditions and can then explore how future conditions may lead to shifts in these same DPFs in the future. We analyze DPFs in the context of *I. scapularis* and Lyme disease risk, but note that the approach shows promise for other organisms and disease systems.

## 2. Methods

### 2.1. Modeling Methodology

The overarching analysis involved four key steps. First, a deterministic, dynamic population model was run, in parallel, over a large geographic area to generate spatially explicit simulations of population density in response to temperature variation. A daily time step was used in conjunction with the smallest grid cell size for which temperature data were available from a global circulation model. Second, simulated population dynamics were recorded at each grid cell for each vector life stage under current and future climate scenarios, and these were characterized in terms of their dynamic population features (DPFs), which were chosen to highlight population trends, seasonality or a combination of both. Third, DPFs were evaluated for their ability to predict the current distribution of vectors or human disease risk, using publically available data. Finally, DPF values found to be important determinants of current vector or disease distributions were visualized across the spatial domain for a range of future climate scenarios. We describe each of these steps in detail, with the application to Lyme disease, next.

### 2.2. Lyme Model

A twelve-stage temperature-driven life cycle model of black-legged deer ticks (*I. scapularis*) (described in [28]) was adapted for high-performance simulation using Simulink (v. 7.0) and Matlab (R 2011b) and executed across a cluster of 48 nodes. The model was run using daily (rather than monthly) mean temperatures, and the model was coded to be spatially explicit, executed in parallel at each grid cell across a large spatial domain. Temperature drivers shape simulated tick populations through degree-day functions, which model development delays, and through temperature-dependent activity parameters, which model host-seeking behaviors (see Supplementary Information, Table S1). Using a daily time step, model simulations at each cell were carried out over the domain under both the baseline and projected climate periods. Time series outputs, recorded daily, included questing adult (QA), questing nymph (QN), and questing larva (QL) populations. Given the rather large spatial resolution simulated from the perspective of *Ixodes* ecology (e.g., [34,35]), populations do not interact between grid cells (*i.e.*, im/emigration are not modeled).

### 2.3. Domain and Climate Data

Simulation and subsequent analyses were conducted in the eastern United States across a domain of 4 × 4 km grid cells. Customized climate data across this grid were obtained from the Regional Climate Model (Weather Research and Forecasting (WRF) 3.2.1) simulated at the University of Tennessee/Oak Ridge National Laboratory [36]. Daily temperatures at each cell were calculated as an average of daily minimum and maximum temperatures produced by climate simulations for a baseline time period (2001–2004) and two projected scenarios of differing severity (for 2057–2059). Projected scenarios, Representative Concentration Pathway (RCP) 4.5 and RCP 8.5, correspond to a continuous rise in radiative forcing to 4.5 W/m^2^ (moderate scenario) and 8.5 W/m^2^ (severe scenario), respectively, in 2100 [37].

### 2.4. Dynamic Features of Ixodes Population Response to Seasonality Shifts

Dynamic population features, chosen to highlight *Ixodes* population phenology and seasonality, were determined as described in Table 1 for each year. These were used to compare simulated population dynamics for each life stage at the grid cell level for three years of simulation under both the baseline and projected climate conditions. With the exceptions of the *Mean* or *Median*, calculated as the three-year mean or median of the simulated daily population, DPFs were calculated for each year of the simulation period and averaged to produce a final DPF value at each cell for each climate scenario.

Population response DPFs included three-year *Mean* and three-year *Median* populations; the maximum population during each year (*Peak Population*); and the average number of local maxima per year (*Peaks per Year*). A 90-day moving window was used to identify each local peak through the course of the year. In order to identify peaks in the first 90 days of the simulation output, the last 89 days of simulation output were prepended to the output to provide a 90-day window. Similarly, the first 89 days of simulation output were appended to the simulation in order to aid identification of peaks in the final 90 days of the simulation.

Seasonality DPFs, which were defined for each life stage, included two quantifications of season length and one of season timing. One classification of season length, termed *IP to IP*, was defined as the number of days between inflection points on either side of the annual maximum population. Inflection points were defined as changes in the concavity of the loess-smoothed (30-day window) population time series; changes in concavity were determined using a 3-point central difference equation on the smoothed time series. The second season length quantification was defined as the number of days from the annual maximum population to the annual minimum population, termed *Peak to Trough. Wave Angle* may be understood as the relative timing of each cell’s season. To determine *Wave Angle*, continuous wavelet analysis was carried out using a Morlet wavelet [38] for the period of maximum power, ~90.5 days (see Supplementary Information). DPFs combining seasonality and absolute population included *IP Pop* is the number of tick-days during each life stage’s season (that is, the summation of the tick population for all days included in the *IP to IP* calculation). Additionally, *UQ/IQR* is used to estimate the period within the calendar year where the highest quartile simulated populations occur. Thus, *UQ/IQR* is defined by selecting the time points (days) in which the upper quartile populations occur, then taking the mean of the interquartile range of these time points (Table 1).

### 2.5. Comparison of DPFs to Observed Data

DPFs obtained from the model as described above were fit to observed county-level *I. scapularis* presence (coded in three levels as absent, reported and established) and Lyme disease incidence (coded in four levels as none/minimal, low, medium and high) obtained from the Centers for Disease Control and Prevention (CDC) [33,39]. For all analyses, the four reported classifications were grouped into all possible dichotomizations (e.g., for Lyme disease, dichotomizations included minimal/none *vs.* low, medium and high; minimal/none and low *vs.* medium and high; and minimal/none, low and medium *vs.* high). DPFs were spatially averaged to the county level and compared to the observed (CDC) data using both area under the receiver operating characteristic curve (AUC) and logistic regression to ascertain each DPF’s predictive ability. AUC (range: 0 to 1) quantizes model predictive accuracy for a dichotomous outcome, where a value of 0.5 indicates no predictive ability, a value of 1 indicates perfect discrimination and a value of 0 indicates lack of discrimination. To assess potential spatial variation in the ability of DPFs to predict Lyme disease risk, AUCs for selected DPFs were also determined for counties in three regions (Northeast, South and Midwest), as defined by the US census.

### 2.6. Spatial Sensitivity Analysis

To assess DPF sensitivity to spatial autocorrelation, the above-defined model outputs were also fit to observed Lyme disease and tick prevalence data using both a (non-spatial) logistic model and a spatial logistic model. The spatial regression model is defined as follows:

$$\text{logit}[P(y_{i}=1)]=\alpha +\lambda_{i}+\beta X_{i}$$

where *y_i_* is the dichotomized observed Lyme disease category, α is the overall baseline risk, λ_*i*_ is the county-specific spatial random effects and β represents the log odds ratio associated with DPFs of population response (*X_i_*). The model used controls for the effects of spatial autocorrelation using an intrinsic conditional autoregressive (CAR) model [40]. The CAR model, often formulated by the conditional distribution of λ_*i*_, given its neighbors, assumes that λ_*i*_, for each county, *i*, is a spatial average of its neighbors. The conditional distribution is Gaussian, with mean $\frac{1}{m_{i}}\sum_{i\sim i\prime }\lambda_{i}$ and variance τ^2^/*m_i_*, where *i~i*′ denotes that county, *i*, shares a boundary with county *i*′, and *m_i_* is the number of boundary-sharing neighbors for county *i*. The percent change in β and the magnitude of parameter, τ^2^, which controls the degree of spatial similarly, were used to assess the improvement in fit provided by the addition of the spatial term to the logistic regression model.

## 3. Results

Simulated population dynamics for the three questing tick life stages were obtained from a temperature-forced model simulated across a grid of 4 × 4 km cells covering the eastern United States under both baseline and projected climate. Based on these simulated population dynamics, ten dynamic population features (DPFs) were derived and analyzed spatially to characterize the population response to current and future climate across the domain. Pairwise correlations between DPFs were determined, and each DPF under the current climate was assessed for its ability to discriminate Lyme disease risk and vector presence/absence using observed data from the US CDC.

### 3.1. Correlation among DPFs

To determine the degree to which DPFs are collinear and, thus, to quantify related aspects of the population curve, correlations were calculated for all pairs of DPFs. There was strong correlation between two different DPFs of absolute population (*Mean* and *Median*) in all questing life stages over the spatial extent of analysis (r_s_ ≥ 0.96; see Table 2). *Peaks per Year* and *IP Pop* were also highly correlated (r_s_ ≥ 0.88) with absolute population DPFs and each other in the questing adult stage, while *Peaks per Year* was also highly correlated (r_s_ ≥ 0.90), with absolute population DPFs and *IP to IP* in the questing nymph and larval stages. Only *Wave Angle* was inversely correlated with all other DPFs in the nymph and adult stages. With the exception of its correlation with *UQ/IQR* (QA: r_s_ = −0.77; QN: r_s_ = −0.81), this inverse relationship was weak (r_s_ ≥ −0.63). In the questing larval stage, only *IP to IP* showed a weak inverse correlation with other DPFs (save for its correlation with *UQ/IQR*). No timing DPF was strongly associated with any other timing DPF in any life stage.

### 3.2. Comparison of DPFs to Observed Data

Each DPF was fit to observed tick presence or to Lyme disease risk from the US CDC, producing an AUC value, a discriminatory index that allows comparison of continuous predictions to dichotomous observations without requiring subjective cut points. *Peak Month* and *Peak Population* showed the greatest discriminatory ability across all life stages when compared with CDC Lyme disease risk data (Table 3; AUC = 0.54 to 0.90). Among most DPFs, the minimal *vs.* high or the minimal/low/medium *vs.* high dichotomizations gave higher AUC values than the remaining dichotomizations. The dichotomization of minimal *vs.* low/medium/high performed worst in AUC analyses across all questing life stages. *Peak to Trough, IP to IP, IP Pop, UQ/IQR*, and *Wave Angle* showed inconsistent predictive ability over all three life stages. Of these, only *IP Pop* in the QN life stage showed some improved discriminatory ability.

Comparison of DPFs to CDC tick presence data showed markedly less discriminatory ability. *Peak Population, Peak Month, Peak to Trough* and *UQ/IQR* were statistically significant predictors of tick presence across all questing life stages and dichotomizations of CDC data, but AUC values were low and ranged from 0.53 to 0.71 for QA, from 0.54 to 0.69 for QN, and from 0.52 to 0.69 for QL. For QL *Wave Angle* and tick presence, AUC ranged from 0.66 to 0.7. Along with QA *Peak Month* and QA *Peak Population*, this was among the strongest predictors of tick presence. However, for the dichotomization minimal/none *vs.* low/medium/high, DPFs, *Mean, Median, Peaks per Year, IP to IP, IP Pop* and *UQ/IQR*, were uniformly non-significant across all questing life stages. *Peak Month* AUC values were very similar for QN and QL, and slightly lower for QA.

#### 3.2.1. Regional Analyses

A regional analysis, analogous to the preceding AUC analysis, was carried out for *Peak Month* and *Peak Population*, to ascertain the degree to which discriminatory ability varies by location. In most cases, AUCs for *Peak Month* and *Peak Population* were observed to be highest (Table 4) in the Midwest region of the eastern United States (AUC: 0.80 to 0.96), where the AUC was most often statistically significant. AUC values in the North were consistently lower than the Midwest (AUC: 0.71 to 0.78), for both DPFs. In the South, *Peak Month* usually demonstrated higher statistically significant predictive ability for QN and QL than *Peak Population,* while both DPFs demonstrated similar predictive ability for QA.

#### 3.2.2. Spatial Sensitivity Analysis

Regression coefficients produced in conditional autoregressive models for each DPF at each life stage on CDC data were not substantively different from those produced by a non-spatial regression (not shown). Conditional autoregressive models exhibited low values of (0.003, 0.06) relative to the intercept and/or the parameter value, indicating that the contribution of spatial autocorrelation is small.

### 3.3. Shifts in Geographic Distribution of DPFs

**Figure 1:**
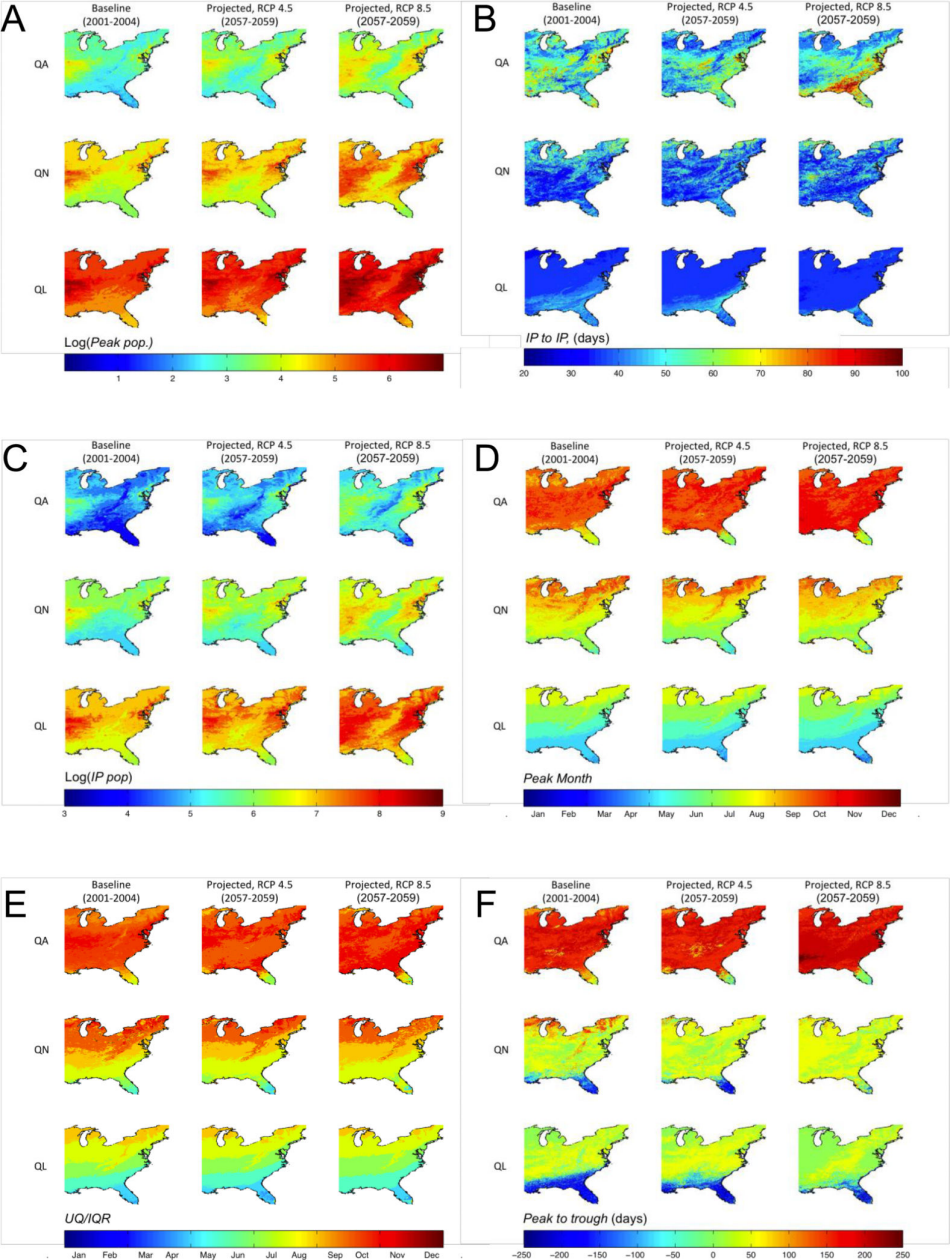
(**A**) Log of *Peak Population*, (**B**) *IP to IP*, (**C**) log of *IP Pop*, (**D**) *Peak Month*, (**E**) *UQ/IQR* and (**F**) *Peak to Trough* for questing adults (QA), questing nymphs (QN) and questing larvae (QL).

DPFs in all cells (N = 262,152) under future climate scenarios were significantly (*p* < 0.001) different than those from baseline simulations. Simulated mean, median and peak populations all show increases across most of the eastern United States, with the largest increases in the RCP 8.5 scenario. In particular, 18.8%, 7.7% and 4.1% of cells showed an increase of an order of magnitude or greater in peak QA, QN and QL population, respectively, in the RCP 8.5 scenario. Relative to baseline, regions of highest projected mean, median and peak tick population expanded both northward and southward to encroach upon the areas of low DPF values occurring across the Appalachian mountain range (e.g., Figure 1).

While both projected scenarios showed simulated questing life stage average *Peaks per Year* that were significantly different from the baseline case and the two projected scenarios were significantly different from one another, there was no substantive change (<0.01 peaks per year) when these comparisons were made across the entire domain. Variations in population response between scenarios, as shown by *Peaks per Year*, demonstrate the lack of uniform response (Figure 1). The number of cells projected to have two or more population peaks per year for the QA life stage increased in northern regions, while there was a net decrease in the number of cells with more than one peak in southern regions. In contrast, for the QL and QN life stages, the number of cells experiencing more than one population peak per year decreased uniformly across the spatial domain.

Under scenarios RCP 4.5 and RCP 8.5 compared to baseline, the season length for QA, as defined by *IP to IP*, has a pronounced increase from 50–70 day seasons to 60−90 and 80−100 day seasons, respectively, in the South. In the North, there is a net decrease in QA season length for both future scenarios (Figure 1). For QN, projected season length remained the same as baseline, though there was a slight decrease in season length for much of the northern portion of the study area in both projected scenarios, and an increase of approximately 40 days in season length in a small portion of the land-locked Midwest. Projected QL season length showed a decrease of approximately 10 days in southern areas, while the overall geographic area with higher season lengths decreased with increasing scenario severity. Changes in projected season length, as defined by *Peak to Trough*, were approximately uniform across the domain (not shown). QA *Peak to Trough* lengthened on average by eight days in RCP 4.5, and by 1.4 days in RCP 8.5, as compared to baseline. QN and QL *Peak to Trough* shortened by 56.6 and 31.1 days, respectively, in the RCP 8.5 scenario, and by 15 and 10 days, respectively, in the RCP 4.5 scenario.

The exposure DPF, *IP Pop*, which counts the number of tick-days during a season bounded by inflection points around the yearly maximum population, bears little similarity to *IP to IP* maps (Figure 1). *IP Pop* increases in exposure-time across the domain as the severity of the projected scenarios increases. The regions with the highest baseline number of ticks, present during the inflection point defined season, spread outward in both the north and south direction and center around the Midwest and the Northeast.

*Wave Angle* results (see Supplementary Information; Figure S1) showed that the dynamics of all life stages under both projected climate conditions lag behind those at baseline climate by ≤4 days across the simulated domain. The projected month of peak population (*Peak Month*) and *UQ/IQR* for QA generally shifted to earlier months. However, with increasing scenario severity, QN and QL generally shifted to later months across the geographic area.

*Wave angle* results (see Figure S1) showed that the dynamics of all life stages under both projected climate conditions lag behind those at baseline climate by ≤4 days across the simulated domain. The projected month of peak population (*Peak Month*) and *UQ/IQR* for QA generally shifted to earlier months. However, with increasing scenario severity, QN and QL generally shifted to later months across the geographic area.

## 4. Discussion and Conclusions

When examining the response of vector populations to climate change, shifts in phenology, seasonality and other dynamic characteristics can be anticipated across the spatial range and life stages of the organism of interest. Risk of VBD is dependent on both timing and probability of exposure to the vector, and thus, characterizing the dynamic population response over space is crucial in order to anticipate and manage potential future risks. Here, we provide a framework for evaluating both static and dynamic effects of climate change on populations over large geographic areas, using spatially explicit simulation of a climate-driven, stage-structured population model.

Our findings with respect to *Ixodes scapularis* illustrate both the methodology and its utility. The derivation and analysis of dynamic population features are key to the analytical approach. DPFs provide quantitative information about a range of population characteristics and allow for comparison between dynamic simulation output and observed disease data, as well as between baseline and projected climates. Absolute DPFs, such as *Mean, Median* and *Peak Population* can be interpreted as indicators of survivorship, while timing DPFs, such as number of days from the yearly maximum population to the yearly minimum population (*Peak to Trough*) and month in which peak population occurs (*Peak Month*) characterize the timing and length of a given life stage’s season.

In the case of *Ixodes*, DPFs associated with the peak of the simulated population curve, *Peak Population* and *Peak Month*, proved to be the most important in predicting high risk of Lyme disease, though all DPFs showed some level of discriminatory ability. AUC analyses showed that dichotomizations isolating high risk improved discriminatory ability across all DPFs and life stages. Aggregation of medium and high risk also showed improved discriminatory ability across life stages and DPFs as compared to the minimal *vs.* low/medium/high dichotomization. This trend of improvement, as high disease risk is progressively isolated into a single category, suggests strongly that these DPFs are useful in predicting the timing and location of higher Lyme disease risks.

When DPFs are examined for two projected climate scenarios, we show that the dynamic population response of *I. scapularis* is not uniform across life stages and varies over space. Spatial shifts in temporal features include geographic shifts in season, and these shifts are not consistently northward as one might intuitively hypothesize. While the month in which the greatest number of ticks are questing (*Peak Month*) is delayed for the adult life stage (Figure 1), QN and QL peaks do not show geographically uniform shifts to earlier questing season. Also, *Peak to Trough* and *IP to IP* indicate potential changes in season length in projected scenarios. Spatial shifts in absolute DPFs, such as *Peak Population*, vary by region. For instance, the peak populations in the Midwest and the Northeast regions are both expected to rise far more as compared to the Appalachian mountain range or the Gulf Coast, where these populations are expected to remain more stable.

Although the finding that QL *Peak Month* and *Peak Population* show high predictive ability for Lyme disease risk is significant, the causal implications of this finding, and others like it, must be interpreted cautiously. Disease risk is not directly related to the questing larval stage, which takes the first blood meal in the lifecycle, and thus, is responsible for Lyme transmission only under the rare circumstance that larvae are infected transovarially. Likewise, QN *Peak Month* and *Peak Population* have similar AUC values for all dichotomizations of Lyme disease risk, an effect driven largely by the similarity of tick response to temperature in these two life stages, rather than mutual causal relationships with disease. Complex temporal relationships are inherent in these populations: questing nymphs and questing larvae, for instance, peak at approximately the same time of year, and their populations in a given location are ostensibly correlated, though the QN population does not result from the QL population in the same year, but rather previous years’ QL.

As in other ecological modeling analyses, data quality determines the utility of this analysis framework for a given system. In our analysis, CDC data quality may account for the lack of significant AUCs of DPFs in comparison to the observed tick data. Tick presence/absence data are collected using a variety of methods, such as dragging and deer surveys, often under serious resource constraints [33]. Rather than providing consistent, systematic information about tick presence and absence, the national tick dataset offers a coarse categorization derived from disparate information. This is in contrast to the national Lyme disease dataset, which is based on a consistent reporting standard. Given the higher quality of data collected, this dataset is more useful in substantiating the results of our model.

Other climate factors besides temperature, such as humidity, have been shown to affect *Ixodes* spp*.* activity [41,42] and correlate with human Lyme disease risk [43]. The population model used here did not incorporate *Ixodes*’ response to humidity, and although our simulated population data demonstrated good correspondence with Lyme incidence, it is possible that including other key environmental variables may yield yet greater correspondence. Likewise, host and pathogen populations were not considered in our analysis, which was limited to vector dynamics. Relatively little research has been done on the potential population responses of *Borrelia* spp*.* under altered climate conditions. However, it has been suggested that changes in *Ixodes* phenology in response to climatic changes may affect the evolution of various tick-borne pathogens, so as to modify their lifespan, transmission and pathogenicity [44]. Host dynamics can also greatly impact infected vector density and consequent human risk in a variety of VBD systems [45–47]. In the case of Lyme disease, the abundance of key hosts, such as mice and chipmunks, has been shown to predict the density of infected nymphs in eastern deciduous forests [48]; in other areas, such as the southern United States, lizards are believed to exert a dampening effect on the spread of Lyme disease, due to poor host competence or zooprophylactic effects [49,50]. Including host, vector and pathogen dynamics in a combined model would pose significant methodological and computational challenges, but is also likely to add greatly to our mechanistic understanding of shifting VBD risk under future environmental conditions. We note that a similar simulation, summarization (e.g., DPFs) and analysis approach can be pursued with such a combined model; yet, other summarizations (e.g., *R*_0_) become available for geovisualization in that context (e.g., [51,52]).

The methodological contributions made by the modeling analysis described here are considerable. We provide a quantitative assessment of population dynamics—with potential consequences for disease risk—under future climates, which is made possible by use of a spatially-explicit, mechanistic model [53]. Our spatial characterization of DPFs allows a detailed visual assessment (e.g., Figure 1), alongside a quantitative analysis, of the dynamic population response to future climate, revealing potential changes that are non-intuitive. For instance, across the eastern United States, under projected temperatures as compared to the baseline scenario, nymphs and larvae are projected to arrive at their peak population earlier in the season, while adults are projected to reach peak population later in the season (Figure 1). The approach taken here also highlights the value of modeling abundance, which, unlike habitat suitability or other static measures, allows for the examination of phenology and seasonality among life stages and the potential implications for (and correlation with) disease risk. For instance, *IP to IP* indicates that the length of larval “season” is stable across the three temperature scenarios, while the adult, and, to a lesser extent, the nymphal stages exhibit “seasons” that are strongly sensitive to the projected increasing temperatures. Such life-stage-specific responses in time and space would be unapparent using traditional methods that examine, for instance, aggregate, annual effects.

We caution above against a causal interpretation of a DPF’s predictive power. A strong correlation between a DPF and observed disease incidence may not represent a causal relationship, but such a finding can raise hypotheses that ultimately lead to greater mechanistic understanding of the relationship between vector populations and disease risk in space and time and, thus, an improved causal understanding. Finally, population models, such as the one examined here, can also be used to evaluate the efficacy and economy of potential public health interventions [53], such as vector or host control (e.g., [54–56] for Lyme disease). A coupled analysis of the effect of temperature in the presence of a vector control program would be an obvious extension of the approach, and such an application of this model is possible for many different vectors, interventions and diseases.

We have demonstrated the ability of a spatially-explicit dynamic population model to discriminate between dynamic population features most strongly associated with disease risk, as well as to characterize the geographically varied response of *I. scapularis* life stages to climate dynamics. Use of such an approach to describe shifts in dynamics is not limited to Lyme disease. The technique may provide new insights into the dynamic responses of a range of disease vectors to environmental changes, particularly shifts in their seasonal and phenological features. Such analyses may provide helpful information about the consequent risk of vector-borne disease under future conditions.

## Supplementary Material

Supplementary info

## Figures and Tables

**Table 1:** Dynamic population features (DPFs) of population response.

***Absolute Population Features***		
**Mean &** **Median**	Avg. and median population (3yr)	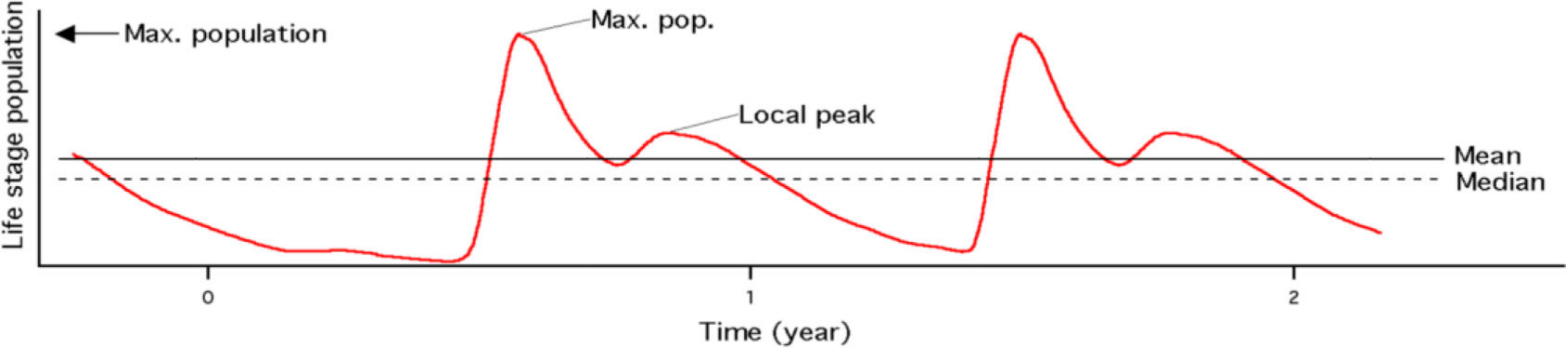
**Peak Pop.**	Avg. of maximum yearly population	
**Peaks per** **Year**	Avg. no. of peaks per year	
***Timing Population Features***		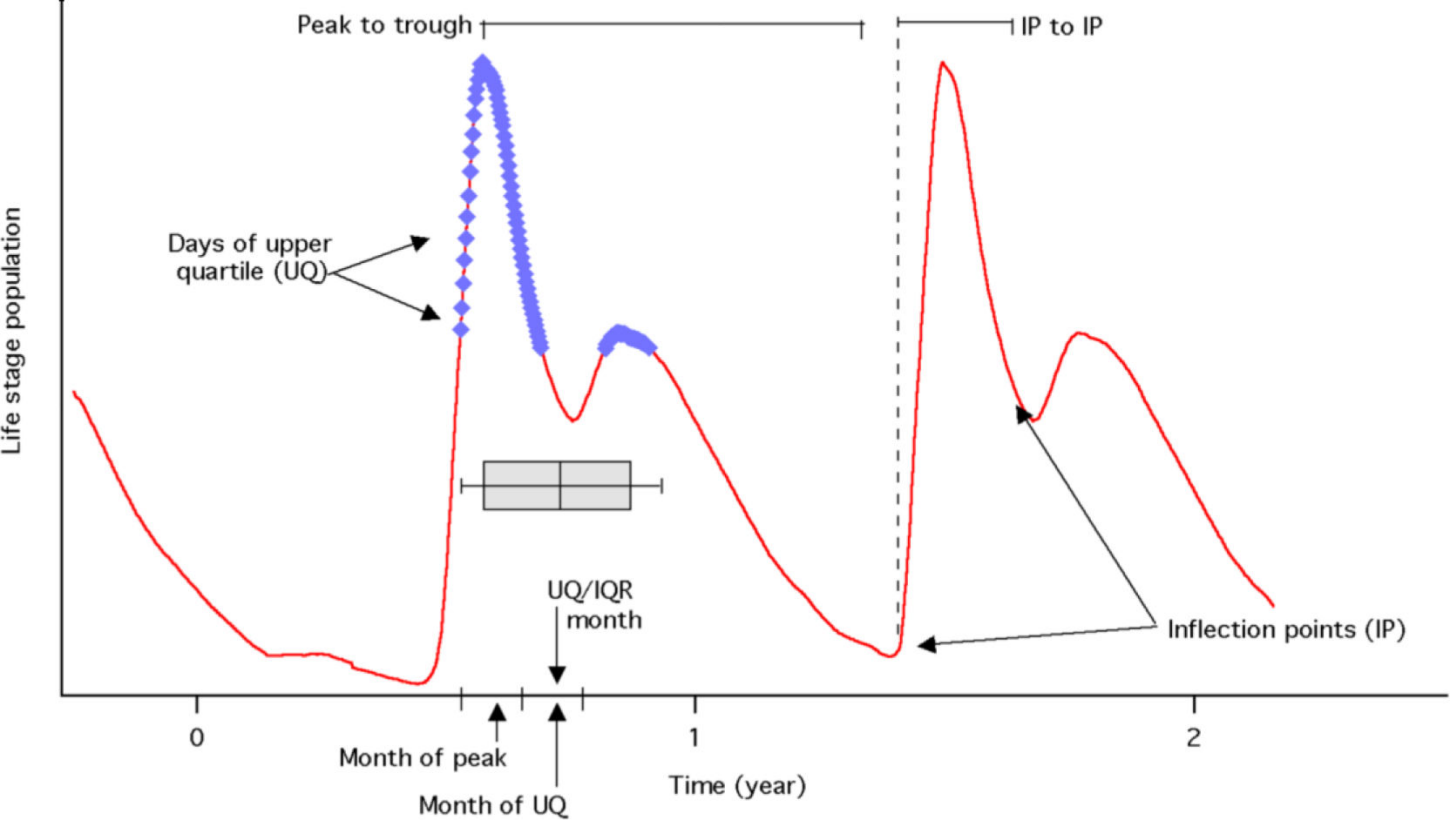
**Peak** **Month**	Month of the yearly peak	
**Peak to** **Trough**	No. of days between yearly peak and yearly trough	
**IP to IP**	Time between inflection points (IP) on either side of yearly max. pop.	
**UQ/IQR**	Avg. of month during which the inter-quartile range (IQR) of the upper quartile (UQ) occur	
**Wave** **Angle**	Wave angle for period = 90.5 days, from continuous wavelet analysis using a complex Morlet waveform (after [33]).	
***Exposure Population Features***		
**IP Pop**	The summation of tick population for all days included in the IP to IP calculation	

**Table 2:** Spearman correlation coefficients (rs) * were assessed between DPFs at each cell for each questing life stage under the baseline climate scenario.

	Mean	Median	Peak Population	Peaks per Year	Peak Month	Peak to Trough	IP to IP	IP Pop	UQ/IQR	Wave Angle
**Questing Adult**										

**Mean**	1	0.98	−0.48	0.99	−0.47	0.25	0.26	0.98	0.55	−0.34
**Median**	0.98	1	−0.47	0.96	−0.46	0.24	0.19	0.94	0.47	−0.24
**Peak** **Population**	−0.48	−0.47	1	−0.46	0.86	0.11	−0.17	−0.48	−0.11	0.10
**Peaks per** **Year**	0.99	0.96	−0.46	1	−0.44	0.26	0.29	0.99	0.60	−0.38
**Peak Month**	−0.47	−0.46	0.86	−0.44	1	0.08	−0.19	−0.47	−0.14	0.08
**Peak to** **Trough**	0.25	0.24	0.11	0.26	0.08	1	0.09	0.23	0.47	−0.33
**IP to IP**	0.26	0.19	−0.17	0.29	−0.19	0.09	1	0.40	0.48	−0.38
**IP Pop**	0.98	0.94	−0.48	0.99	−0.47	0.23	0.40	1	0.63	−0.41
**UQ/IQR**	0.55	0.47	−0.11	0.60	−0.14	0.47	0.48	0.63	1	−0.77
**Wave Angle**	−0.34	−0.24	0.10	−0.38	0.08	−0.33	−0.38	−0.41	−0.77	1

**Questing Nymphs**										
**Mean**	1	1.00	−0.42	0.99	0.20	0.17	0.99	0.37	0.60	−0.45
**Median**	1.00	1	−0.41	0.98	0.21	0.18	0.98	0.38	0.62	−0.47
**Peak** **Population**	−0.42	−0.41	1	−0.43	−0.59	−0.01	−0.43	−0.11	−0.10	0.02
**Peaks per** **Year**	0.99	0.98	−0.43	1	0.19	0.14	0.99	0.35	0.55	−0.40
**Peak Month**	0.20	0.21	−0.59	0.19	1	−0.25	0.17	0.12	0.03	0.17
**IP to IP**	0.17	0.18	−0.01	0.14	−0.25	1	0.26	0.49	0.58	−0.63
**IP Pop**	0.99	0.98	−0.43	0.99	0.17	0.26	1	0.40	0.61	−0.46
**Peak to** **Trough**	0.37	0.38	−0.11	0.35	0.12	0.49	0.40	1	0.71	−0.51
**UQ/IQR**	0.60	0.62	−0.10	0.55	0.03	0.58	0.61	0.71	1	−0.82
**Wave Angle**	−0.45	−0.47	0.02	−0.40	0.17	−0.63	−0.46	−0.51	−0.82	1

**Questing Larvae**										

**Mean**	1	0.96	−0.43	0.98	0.20	−0.52	0.97	0.50	0.55	0.36
**Median**	0.96	1	−0.37	0.90	0.17	−0.65	0.88	0.44	0.70	0.27
**Peak** **Population**	−0.43	−0.37	1	−0.43	−0.57	0.17	−0.43	0.03	−0.09	0.13
**Peaks per** **Year**	0.98	0.90	−0.43	1	0.20	−0.44	0.99	0.55	0.46	0.45
**Peak Month**	0.20	0.17	−0.57	0.20	1	−0.11	0.19	−0.03	0.00	−0.06
**IP to IP**	−0.52	−0.65	0.17	−0.44	−0.11	1	−0.39	−0.38	−0.92	−0.16
**IP Pop**	0.97	0.88	−0.43	0.99	0.19	−0.39	1	0.55	0.42	0.46
**Peak to** **Trough**	0.50	0.44	0.03	0.55	−0.03	−0.38	0.55	1	0.41	0.74
**UQ/IQR**	0.55	0.70	−0.09	0.46	0.00	−0.92	0.42	0.41	1	0.17
**Wave Angle**	0.36	0.27	0.13	0.45	−0.06	−0.16	0.46	0.74	0.17	1

* All values are significant at *p* < 0.0005.

**Table 3:** Area under the receiver operating characteristic curve (AUC) analysis comparing dynamic population features (DPFs) to observed Lyme disease incidence and tick presence.

Observational Data Set / Dichotomization	N	Mean	Median	Peak Population	Number Peaks/Yr	Peak Month	Peak to Trough	IP to IP	IP Pop	UQ/ IQR	Wave Angle
**Questing Adults**											

Lyme disease risk											
Minimal *vs.* Low/Medium/High	1,683	0.47	0.48	**0.73**	0.45	**0.70**	**0.60**	**0.56**	0.45	**0.62**	**0.51**
Minimal/Low *vs.* Medium/High	1,683	**0.64**	**0.67**	**0.81**	**0.62**	**0.82**	0.53	0.48	**0.60**	**0.58**	**0.59**
Minimal/Low/Medium *vs.* High	1,683	**0.72**	**0.74**	**0.83**	**0.72**	**0.84**	**0.74**	**0.63**	**0.71**	**0.68**	**0.61**
Minimal *vs.* High	844	**0.71**	**0.73**	**0.90**	**0.70**	**0.89**	**0.69**	**0.61**	**0.68**	**0.63**	**0.60**
Tick presence											
None *vs.* Reported/Established	1,683	0.52	0.52	**0.69**	0.54	**0.67**	**0.60**	**0.54**	0.54	**0.61**	**0.53**
None/Reported *vs.* Established	1,683	0.48	0.49	**0.67**	0.47	**0.67**	**0.53**	0.52	0.46	**0.57**	0.52
None *vs.* Established	1,305	0.47	0.49	**0.71**	0.46	**0.70**	**0.56**	0.53	0.45	**0.60**	**0.52**

**Questing Nymphs**											

Lyme disease risk											
Minimal *vs.* Low/Medium/High	1,683	0.46	0.45	**0.70**	0.46	**0.54**	**0.57**	**0.53**	0.47	**0.60**	**0.56**
Minimal/Low *vs.* Medium/High	1,683	**0.70**	**0.70**	**0.79**	**0.68**	**0.76**	**0.61**	**0.71**	**0.71**	**0.76**	**0.79**
Minimal/Low/Medium *vs.* High	1,683	**0.75**	**0.74**	**0.80**	**0.75**	**0.90**	0.40	0.56	**0.75**	**0.61**	**0.69**
Minimal *vs.* High	844	**0.73**	**0.73**	**0.86**	**0.73**	**0.92**	0.65	**0.56**	**0.74**	**0.55**	**0.67**
Tick presence											
None *vs.* Reported/Established	1,683	0.53	0.53	**0.67**	0.53	**0.55**	**0.54**	**0.54**	0.52	**0.55**	**0.51**
None/Reported *vs.* Established	1,683	0.49	0.49	**0.65**	0.49	**0.69**	**0.58**	**0.54**	0.50	**0.53**	0.48
None *vs.* Established	1,305	0.48	0.52	**0.69**	0.48	**0.68**	**0.58**	**0.55**	0.49	**0.54**	**0.49**

**Questing Larvae**											

Lyme disease risk											
Minimal *vs.* Low/Medium/High	1,683	0.46	0.54	**0.70**	0.45	**0.54**	**0.73**	**0.58**	0.46	**0.58**	**0.75**
Minimal/Low *vs.* Medium/High	1,683	**0.69**	**0.73**	**0.79**	**0.66**	**0.76**	**0.52**	**0.75**	**0.65**	**0.78**	**0.46**
Minimal/Low/Medium *vs.* High	1,683	**0.74**	**0.72**	**0.80**	**0.75**	**0.90**	**0.52**	**0.62**	**0.73**	**0.63**	**0.58**
Minimal *vs.* High	844	**0.73**	**0.71**	**0.85**	**0.72**	**0.92**	0.37	**0.58**	**0.71**	**0.61**	0.58
Tick presence											
None *vs.* Reported/Established	1,683	0.53	0.53	**0.68**	0.54	**0.55**	**0.66**	**0.55**	0.53	**0.54**	**0.70**
None/Reported *vs.* Established	1,683	0.49	0.49	**0.65**	0.48	**0.69**	**0.65**	**0.53**	0.48	**0.52**	**0.66**
None *vs.* Established	1,305	0.52	0.52	**0.69**	0.47	**0.68**	**0.69**	**0.55**	0.47	**0.53**	**0.71**

* Bold indicates significance; orange indicates AUC > 0.8 and *p* < 0.05; blue indicates 0.8 > AUC > 0.7 and *p* < 0.05.

**Table 4:** Regional AUC * sub-analyses.

	Midwest			North			South		
	
Observational Data Set/Dichotomization	N	Peak Month	Peak Population	N	Peak Month	Peak Population	N	Peak Month	Peak Population
**Questing Adults**									

Lyme disease risk									
Minimal *vs*. Low/Medium/High	461	**0.82**	**0.81**	214	0.68	0.67	1,008	**0.60**	0.64
Minimal/Low *vs*. Medium/High	461	**0.81**	**0.80**	214	**0.75**	**0.73**	1,008	**0.72**	**0.71**
Minimal/Low/Medium *vs*. High	461	**0.89**	**0.92**	214	**0.72**	**0.71**	1,008	**0.70**	**0.70**
Minimal *vs*. High	226	**0.95**	**0.96**	88	**0.79**	**0.78**	530	**0.77**	**0.78**

**Questing Nymphs**									

Lyme disease risk									
Minimal *vs*. Low/Medium/High	461	**0.82**	**0.81**	214	0.66	0.67	1,008	**0.53**	**0.62**
Minimal/Low *vs*. Medium/High	461	**0.81**	**0.80**	214	**0.75**	**0.74**	1,008	**0.95**	**0.63**
Minimal/Low/Medium *vs*. High	461	**0.91**	0.91	214	**0.72**	**0.72**	1,008	**0.94**	0.62
Minimal *vs*. High	226	**0.96**	0.96	88	**0.78**	**0.78**	530	**0.97**	**0.78**

**Questing Larvae**									

Lyme disease risk									
Minimal *vs*. Low/Medium/High	461	**0.82**	**0.81**	214	0.66	0.63	1,008	0.53	**0.62**
Minimal/Low *vs*. Medium/High	461	**0.81**	**0.80**	214	**0.75**	**0.71**	1,008	**0.95**	**0.61**
Minimal/Low/Medium *vs*. High	461	**0.91**	0.90	214	**0.72**	**0.71**	1,008	**0.94**	0.58
Minimal *vs*. High	226	0.96	0.96	88	**0.78**	**0.76**	530	**0.97**	**0.63**

* Bold indicates significance; orange indicates AUC > 0.8 and *p* < 0.05; blue indicates 0.8 > AUC > 0.7 and *p* < 0.05.
