# Supplementary material for: Spatially-Explicit Simulation Modeling of Ecological Response to Climate Change: Methodological Considerations in Predicting Shifting Population Dynamics of Infectious Disease Vectors
Source: ISPRS Int J Geoinf. Author manuscript; Available in PMC 2014 Apr 23. (PMC3997168; doi:10.3390/ijgi2030645)
Supplement: Supplementary info [file NIHMS542944-supplement-Supplementary_info.docx]

**OPEN ACCESS**

ISPRS International
Journal of **Geo-Information**

**ISSN 2220-9964**

Supplementary Information

Spatially-Explicit Simulation Modeling of Ecological Response to Climate Change: Methodological Considerations in Predicting Shifting Population Dynamics of Infectious Disease Vectors, *ISPRS Int. J. Geo-Inf.* 2013, *2*, 645-664

Radhika Dhingra ^1^, Violeta Jimenez ^1^, Howard H. Chang ^2^, Manoj Gambhir ^3^, Joshua S. Fu ^4^,
Yang Liu ^1^ and Justin V. Remais ^1,5^*

^1^ Department of Environmental Health, Rollins School of Public Health, Emory University,
1518 Clifton Rd. NE, Atlanta, GA 30322, USA; E-Mails: rdhingr@emory.edu (R.D.);
violeta.jimenez@emory.edu (V.J.); yang.liu@emory.edu (Y.L.)

^2^ Department of Biostatistics and Bioinformatics, Rollins School of Public Health, Emory University, 1518 Clifton Rd. NE, Atlanta, GA 30322, USA; E-Mail: howard.chang@emory.edu

^3^ MRC Centre for Outbreak Analysis and Modeling, Department of Infectious Disease Epidemiology, Imperial College London, London, SW7 2AZ, UK; E-Mail: m.gambhir@imperial.ac.uk

^4^ Department of Civil and Environmental Engineering, University of Tennessee, Knoxville,
62 Perkins Hall, Knoxville, TN 37996, USA; E-Mail: jsfu@utk.edu

^5^ Program in Population Biology, Ecology and Evolution, Graduate Division of Biological and Biomedical Sciences, Emory University, 1510 Clifton Rd., Atlanta, GA 30322, USA

* Author to whom correspondence should be addressed; E-Mail: justin.remais@emory.edu;
Tel.: +1-404-712-8908; Fax: +1-404-727-8744.

Wavelet Analysis

The wavelet phase angle was determined for each life stage’s population—each day at each grid cell—by convolution of the population time series with the Morlet wavelet [37] for the period of 90.5 days (which was the period of maximum power for 87.6%–97.0% of cells). Using the wavelet phase angle determined for tick populations in an arbitrary reference location (41.3159°N, 72.3290°W; Lyme, Connecticut), *Wave Angle* expresses the phase difference between the annual averaged wavelet phase angle at a given cell, and at the reference location, where 360 degrees is equivalent to the 90.5 day period. *Wave Angle* at a given cell can thus be interpreted as the phase delay, in time, between the population dynamics at the cell and the equivalent dynamics at the reference cell. Projected *Wave Angle* for QN shows a uniform lag across the eastern United States of, at most, 4 days behind the population dynamics at the reference site, while baseline *Wave Angle* for QN is either concurrent or precedes population dynamics at the reference (Figure S1). Projected QA *Wave Angle* shows an increased lag of up to 2 days behind the reference location in patches primarily in the Southern portion of the domain as compared to the in-phase dynamics indicated by the baseline QA *Wave Angle*.

**Figure S1.** *Wave Angle* (period = 90.5 days) for questing adults (QA), questing nymphs (QN) and questing larvae (QL). The DPF, *Wave Angle,* is calculated at each cell in comparison to an arbitrary reference location (see text).

**Table S1.** Temperature-dependent delay development terms and activity coefficients for the *I. scapularis* 12 stage life stage model [1].

| **Parameter** | **Definition** | **Parameter Formulation or Temperature Range (°C)** |
| --- | --- | --- |
| q | Time delay for egg pre-eclosion period | 34,234 × (Temperature)^−2.27^ |
| s | Time delay for engorged larva to nymph development | 101,181 × (Temperature)^−2.55^ |
| v | Time delay for engorged nymph to adult development | 1,596 × (Temperature)^−1.21^ |
| x | Time delay for the pre-oviposition period | 1,300 × (Temperature)^−1.42^ |
| θ_i_ | Temperature-variable factor for questing activity of nymphs and larvae | (11, >30) |
| θ_a_ | Temperature-variable factor for questing activity of adults | (3, 16) |

References

1. Chuine, I.; Beaubien, E.G. Phenology is a major determinant of tree species range. *Ecol. Lett.* **2001**, *4*, 500–510.

© 2013 by the authors; licensee MDPI, Basel, Switzerland. This article is an open access article distributed under the terms and conditions of the Creative Commons Attribution license (http://creativecommons.org/licenses/by/3.0/).
